# Empirical use of growth hormone in IVF is useless: the largest randomized controlled trial

**DOI:** 10.1093/humrep/deae251

**Published:** 2024-11-29

**Authors:** Ali Mourad, Wael Jamal, Robert Hemmings, Artak Tadevosyan, Simon Phillips, Isaac-Jacques Kadoch

**Affiliations:** Department of Obstetrics and Gynecology, University of Montreal, Montreal, QC, Canada; Department of Obstetrics and Gynecology, University of Montreal, Montreal, QC, Canada; Ovo Clinic Montreal, Montreal, QC, Canada; Department of Obstetrics and Gynecology, University of Montreal, Montreal, QC, Canada; Ovo Clinic Montreal, Montreal, QC, Canada; Department of Obstetrics and Gynecology, McGill University, Montreal, QC, Canada; Department of Obstetrics and Gynecology, University of Montreal, Montreal, QC, Canada; Ovo Clinic Montreal, Montreal, QC, Canada; Department of Obstetrics and Gynecology, University of Montreal, Montreal, QC, Canada; Ovo Clinic Montreal, Montreal, QC, Canada; Department of Obstetrics and Gynecology, University of Montreal, Montreal, QC, Canada; Ovo Clinic Montreal, Montreal, QC, Canada

**Keywords:** growth hormone, assisted reproductive technology, pregnancy, *in vitro* fertilization, IVF, embryo, infertility, fertility agents, live birth

## Abstract

**STUDY QUESTION:**

Does adjuvant growth hormone (GH) therapy in GnRH antagonist cycles improve reproductive outcomes in the general IVF population?

**SUMMARY ANSWER:**

Empiric adjuvant GH therapy in GnRH antagonist cycles does not improve IVF stimulation results or reproductive outcomes, including implantation, miscarriage, and clinical pregnancy rates.

**WHAT IS KNOWN ALREADY:**

Previous evidence regarding the benefits of GH therapy in IVF cycles has been inconclusive due to the lack of well-designed, large-scale randomized controlled trials (RCTs) in the general IVF population.

**STUDY DESIGN, SIZE, DURATION:**

This is a phase III open-label RCT involving 288 patients undergoing antagonist IVF cycles at the Ovo clinic in Montreal, Canada, between June 2014 and January 2020. Patients were randomly assigned at a 1:1 ratio to either the GH or control group. The intervention group received daily 2.5 mg subcutaneous injections of GH from Day 1 of ovarian stimulation until the day of oocyte retrieval, while the control group received standard ovarian stimulation without any adjuvant therapy.

**PARTICIPANTS/MATERIALS, SETTING, METHODS:**

Patients were expected normal responders. All embryo transfers, both fresh and frozen, resulting from the studied IVF cycle were included in an intention-to-treat and per-protocol analyses. The primary outcome was the clinical pregnancy rate, while secondary outcomes included the number of retrieved oocytes, good-quality embryos, maturation, fertilization, implantation, and miscarriage rates.

**MAIN RESULTS AND THE ROLE OF CHANCE:**

A total of 288 patients were recruited and randomly assigned at a 1:1 ratio to either the GH or the control group. After excluding cycle cancellations and patients who did not undergo transfer, 105 patients remained in each group. The overall mean age was 38.0 years, the mean BMI was 25.11 kg/m^2^ and the mean anti-Müllerian hormone was 2.51 ng/ml. The cycle characteristics were similar between both groups. No differences were observed regarding the total dose of gonadotropins (4600 versus 4660 IU for the GH and control groups, respectively, *P* = 0.752), days of stimulation (11.4 versus 11.7 days, *P* = 0.118), and endometrial thickness (10.63 versus 10.94 mm, *P* = 0.372). Both the intention to treat (ITT) and per protocol analyses yielded similar results for stimulation outcomes. In the ITT analysis, no differences were found in the number of follicles ≥15 mm (7.8 versus 7.1, *P* = 0.212), retrieved oocytes (11.7 versus 11.2, *P* = 0.613), mature oocytes (8.5 versus 8.6, *P* = 0.851), maturation rate (73.8 versus 78.4%, *P* = 0.060), fertilization rate (64.3 versus 67.2%, *P* = 0.388), and good quality embryos (2.5 versus 2.6, *P* = 0.767). Reproductive outcomes in fresh embryo transfer showed no difference for implantation rate (38.2 versus 39.5%, *P* = 0.829), miscarriage rate (26.5 versus 31.1%, *P* = 0.653), clinical pregnancy rate (43.6 versus 50.0%, *P* = 0.406, rate difference, 95% CI: −0.06 [−0.22, 0.09]), and live birth rate (32.1 versus 33.3%, *P* = 0.860). The number of embryos needed to achieve a clinical pregnancy was 3.0 versus 2.5 in the GH and control groups, respectively. Similarly, reproductive outcomes in first frozen embryo transfer showed no difference for implantation rate (31.6 versus 45.3%, *P* = 0.178), miscarriage rate (28.6 versus 26.3%, *P* = 0.873), clinical pregnancy rate (35.1 versus 44.2%, *P* = 0.406, *P* = 0.356, rate difference, 95% CI: −0.09 [−0.28, 0.10]), and live birth rate (22.8 versus 32.6%, *P* = 0.277). The number of embryos needed to achieve a clinical pregnancy was 3.1 versus 2.4 in the GH and control groups, respectively.

**LIMITATIONS, REASONS FOR CAUTION:**

The study focused on expected normal responders, limiting its applicability to other patient populations such as poor responders.

**WIDER IMPLICATIONS OF THE FINDINGS:**

These findings suggest that adding GH therapy to ovarian stimulation in GnRH antagonist cycles may not benefit the general IVF population. Additional high-quality RCTs are warranted to identify subgroups of patients who might benefit from this treatment.

**STUDY FUNDING/COMPETING INTEREST(S):**

EMD Serono Inc., Mississauga, Canada, supplied Saizen^®^ for the study, free of charge. In addition, they provided funding for the statistical analysis. I-J.K. declares grants or contracts from Ferring Pharmaceuticals, consulting fees from Ferring Pharmaceuticals, honoraria from Ferring Pharmaceuticals and EMD Serono, support for attending meetings or travel from Ferring Pharmaceuticals and EMD Serono, participation on a Data Safety Monitoring Board or Advisory Board for Ferring Pharmaceuticals, and stock or stock options from The Fertility Partners; W.J. declares support for attending meetings or travel from EMD Serono; and S.P. declares stock or stock options from The Fertility Partners. All other authors have no conflicts of interest to disclose.

**TRIAL REGISTRATION NUMBER:**

NCT01715324.

**TRIAL REGISTRATION DATE:**

25 October 2012.

**DATE OF FIRST PATIENT’S ENROLMENT:**

25 June 2014.

## Introduction

IVF protocols are constantly evolving, aiming to lower gonadotropin requirements, enhance follicular recruitment, improve oocyte/embryo outcomes, and overall reproductive results, particularly live birth rate (Barnhart, 2014; Eskew and Jungheim, 2017). Adjuvant therapies have been explored to boost IVF results by modulating controlled ovarian stimulation. These therapies were predominantly used for poor responders due to their treatment challenges and poor prognosis (Nardo and Chouliaras, 2020; Zhang *et al.*, 2020). Growth hormone (GH) was first applied to IVF in human subjects in 1988 (Homburg *et al.*, 1988). Early studies suggested that GH sensitized ovarian response to gonadotropins, thereby reducing stimulation duration and total gonadotropin dose required (Homburg *et al.*, 1988).

GH is a pituitary hormone (Brinkman *et al.*, 2022), but also an autocrine/paracrine regulator of female reproductive function, due to local production in the ovaries and uterus, and the presence of its receptors in granulosa cells, theca cells, oocytes, and the endometrium (Sirotkin, 2005; Hull and Harvey, 2014). This expression of GH helps boost intra-ovarian insulin-like growth factor 1 (IGF-1) production, crucial for luteinizing hormone receptor formation, aromatase activity, oocyte maturation, and the production of estrogen and progesterone (Erickson *et al.*, 1989; Yoshimura *et al.*, 1996; van den Eijnden and Strous, 2007). Recombinant DNA technology allows simple synthesis of GH for human use (Duffy *et al.*, 2010).

Studies suggest GH plays a role in ovarian steroidogenesis, follicular development, and endometrial receptivity (Doldi *et al.*, 1996; Sirotkin *et al.*, 2000; Bachelot *et al.*, 2002; Karamouti *et al.*, 2008; Nakamura *et al.*, 2012; Cui *et al.*, 2019). This has been shown in both human and animal models (Kobayashi *et al.*, 2000; Hassan *et al.*, 2001; de Prada and VandeVoort, 2008; Arunakumari *et al.*, 2010; Magalhães *et al.*, 2011). Research exploring GH’s reproductive function found that GH-deficient women since childhood, had a higher risk of subfertility, pubertal, and menstrual dysfunctions (de Boer *et al.*, 1997). Moreover, genetically engineered mice modified to resist GH suffered reduced fecundity (Bartke *et al.*, 2013).

Clinical Studies assessing GH’s role in IVF cycles present inconsistent results, with low-quality evidence supporting its use among poor responders. A recent Cochrane review revealed a small but significant increase in pregnancy rates and number of oocytes retrieved in the GH group for poor responders (Sood *et al.*, 2021). However, it remained inconclusive on live birth rates. The randomized controlled trials (RCTs) used in this review had high bias with significant heterogeneity, influenced by the inconsistent definitions of poor responders and GH therapy regimens. In terms of normal responders, two earlier RCTs from 1992 found no significant difference in clinical pregnancy rate, number of retrieved oocytes, embryos obtained, or total gonadotropins used for ovarian stimulation (Tapanainen *et al.*, 1992; Younis *et al.*, 1992). Despite a lack of RCTs for the general IVF group, a recent matched case-control study from China including 1562 normal responders with a history of poor embryo quality showed promising results (Liu *et al.*, 2019). In this study, half of the women received GH leading to an increase in the rate of two pro-nuclei, number and quality of embryos, and a thicker endometrium on the trigger day.

The current evidence presents uncertainties about the benefit of empiric use of GH in IVF, due to the absence of well-designed high-quality RCT with an adequate sample size. This study aims to examine the effectiveness of GH therapy in expected normal responders. It is, to our knowledge, the largest RCT conducted on this subject.

## Materials and methods

### Ethics statement

Registered under NCT01715324 in the US clinical trial registry with the following IRB identifier: 2387, this study was reviewed and approved by Health Canada Biologics and Genetics Therapies Directorate and the VERITAS IRB. Investigators clearly explained the study’s purposes to all potential participants. All enrolled patients signed informed consent forms provided by the research and development department. Participants could withdraw from the study at any time, for any reason. This study complied with Health Canada Regulations, ICH good clinical practice guidelines, the Declaration of Helsinki, and adhered to the Consolidated Standards of Reporting Trials (CONSORT) guidelines.

### Study design, subjects, and participants

This was a phase III, single-center, open-label RCT, conducted at Ovo clinic in Montreal, Canada. The primary objective was to assess whether adding daily GH injections (2.5 mg Saizen) to GnRH antagonist cycle significantly improved clinical pregnancy rates compared to those only following the standard antagonist protocol. Clinical pregnancy rate was determined through the detection of a fetal heartbeat at the viability ultrasound between weeks 6 and 8 of gestation.

The study had three secondary objectives:

1. Evaluate the following ovarian stimulation characteristics: total dose of gonadotropins used (IU), number of stimulation days, endometrial thickness, estradiol and progesterone levels on trigger day, cycle cancellation rate (percentage of canceled cycles out of the total number of cycles initiated), and IGF-1 level on trigger day.
2. Assess the following IVF outcomes: number of follicles recruited (≥15 mm and <15 mm), number of retrieved oocytes, number of mature oocytes, oocyte maturation rate (percentage of mature oocytes out of the total number of oocytes retrieved), fertilization rate (percentage of transformation of inseminated or micro-injected oocytes into 2PN), number of available embryos to transfer, number and stage of fresh embryo transfers, number and stage of frozen embryo transfers (FETs), and IGF-1 ratio per utilizable embryo.
3. Examine the following reproductive outcomes: implantation rate (percentage of gestational sacs observed at the viability out of the number of embryos transferred), miscarriage rate (percentage of patients who had a pregnancy loss out of the clinical pregnancies), live birth rate (percentage of live births out of the number of embryos transferred), and the number of embryos needed for a clinical pregnancy.

We recruited women seeking IVF services at Ovo clinic from June 2014 to January 2020. Both treatment and control groups received GnRH antagonist regimens, with a gonadotropin dose individualized based on age, ovarian reserve, weight, and BMI. Participants in the treatment group received a daily 2.5 mg subcutaneous injection of GH (Saizen^®^), provided by EMD Serono, Mississauga, Canada. Saizen^®^ was started with the first day of gonadotropin stimulation to the trigger day. Randomization involved assigning eligible participants to treatment or control groups at a ratio of 1:1 using sequential study numbers. The study included a single IVF cycle per patient, and no blinding was performed.

We included women aged between 30 and 42 years with primary or secondary infertility, undergoing a GnRH antagonist protocol, who had not previously been treated with GH during an IVF cycle, and had an anti-Müllerian hormone (AMH) measurement in the last 24 months. Exclusions were those with a contraindication to GH, BMI ≥35 kg/m^2^, concurrent participation in another trial, AMH <0.5 ng/ml, diabetic, at risk for gestational diabetes, undergoing egg donation, received experimental medication within 3 months, positive HIV/Hepatitis B or C screening, history of recurrent implantation failure (defined as: age 30 to 34 years having had 3 or more day-3 embryos transferred or 2 blastocysts transferred without a positive pregnancy test, or age 35–42 years having had 4 or more day-3 embryos transferred or 3 blastocysts transferred without a positive pregnancy test), inability to communicate in French or English, undergoing endometrial receptivity evaluations, or undergoing pre-implantation genetic testing (PGT).

Data pertaining to the medical history of each participant were collected, including BMI, AMH, antral follicular count (AFC), and early follicular phase serum FSH. Any additional vitamins and supplements used by patients were recorded (Supplementary Table S1). Baseline serum IGF-1 levels were measured on the day of randomization and then on the trigger day. Estradiol and progesterone levels were also measured on the trigger day.

### Clinical management

This study implemented a flexible GnRH antagonist protocol. It began with estradiol priming, using 4 mg of oral Estrace starting on the 21st day of the preceding cycle. This was followed by gonadotropin stimulation beginning on the second day of menstruation. Ultrasound monitoring of follicular development was initiated on Day 6 of stimulation. Once the leading follicle measured ≥14 mm or the serum estradiol (E2) level was ≥2000 pmol/l, a daily 0.25 mg subcutaneous injection of either Cetrorelix (EMD Serono Canada) or Ganirelix (Organon Canada Ltd), was started. When at least three leading follicles were ≥18 mm, the final phase of oocyte maturation was triggered using a 5000-IU subcutaneous injection of urinary hCG (u-hCG). In patients at high risk of ovarian hyperstimulation syndrome, a GnRH agonist trigger, Buserelin (Sanofi-Aventis), was given at 1 mg subcutaneously. In such cases, all embryos were frozen. FETs stemming from the study cycle were analyzed for 3 years after the final participant was recruited. Day 3 cleavage-stage embryos or Day 5 or 6 blastocysts were transferred during either fresh or frozen cycles. Luteal phase support consisted of daily intramuscular injections of 50 mg progesterone starting the night of oocyte retrieval for fresh embryo transfers, or when a trilaminar endometrium measuring ≥8 mm for a medicated FET using transdermal synthetic 17β-estradiol, 200 micrograms/24 h, twice weekly (Estradot patches). Transfers were performed on the morning of the sixth day of progesterone supplementation for blastocysts and the fourth day for cleavage-stage embryos. Only morphologically good-quality embryos were transferred. Modified Gardner score system was used for blastocysts to grade their morphology. As for cleavage-stage embryos, top-quality ones were identified based on having stage-appropriate blastomere numbers and being graded as 1 or 2 according to the Alpha Scientists in Reproductive Medicine and ESHRE Special Interest Group of Embryology (2011).

### Sample size determination and statistical analysis

Sample size was calculated assuming a 50% increase in the clinical pregnancy rate, from around 30% to 45%, which signifies a 15% additional improvement when GH is added. A sample size of 288 participants was needed to achieve a power of 80% with an alpha error of 5%. Statistical analysis was performed using SAS System for Windows version 9.2 software. Categorical data were represented as number and percent, while continuous data were displayed using mean and SD. We used Student’s *t*-test for mean value differences and chi-square analysis for dichotomous variables. Our primary analysis was intention-to-treat (ITT). A per-protocol (PP) sensitivity analysis was conducted for patients who underwent embryo transfer. Reproductive outcomes were compared between the GH and the control groups in two different populations: those undergoing fresh embryo transfer cycles and those having their first cycle of FET. A *P*-value <0.05 was considered statistically significant. The results were also assessed using mean difference with 95% CI for continuous data and rate difference with 95% CI for categorical data. These measurements provide a range within which the true outcome is likely to fall 95% of the time, hence giving a better comprehensive understanding of the significance of the variations observed.

## Results

**Figure 1.**
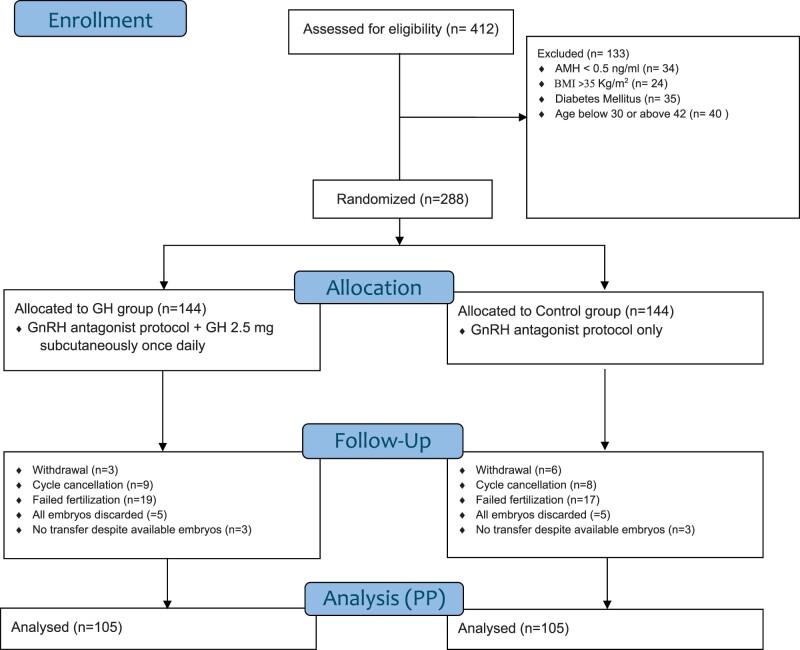
**Consort flow diagram.** AMH, anti-Müllerian hormone; GH, growth hormone, PP, per protocol.

Out of 2380 pre-screened patients, 412 were assessed for eligibility, and 288 were equally and randomly allocated to both study arms. Each group saw 39 dropouts due to factors such as study withdrawal (GH: 3, control: 6), suboptimal response to gonadotropins (GH: 9, control: 8), fertilization failure (GH: 19, control: 17), poor embryo quality (GH: 5, control: 5), freeze all embryos for personal reasons (GH: 3, control: 3). The remaining 105 participants in each group completed their treatment and had an embryo transfer, representing the PP analysis population. The trial’s progress is detailed in the CONSORT flow diagram (Fig. 1).

Both groups had comparable baseline characteristics (Table 1). With an average age of 38 years and a BMI of 25.11 kg/m^2^, the patients were normal responders, as shown by a mean AMH of 2.51 ng/ml, an AFC of 15.19 follicles, and a baseline FSH of 6.63 IU/l. Infertility was primary in 47.6% and secondary in 52.4%, with an average duration of 3.3 years. The causes of infertility were due to female factor (36.8%), male factor (29.2%), mixed male and female factors (9.7%), unexplained infertility (19.4%), and single women/same-sex female couples using donor sperm (4.9%). Almost half of the participants had previously undergone an IVF cycle (47.6%) with the others undergoing their first IVF in this study (52.4%).

**Table 1.** Baseline characteristics of patients according to the intervention (growth hormone [GH]) and control groups.

	GH group (n = 144)	Control group (n = 144)	Overall (n = 288)
Age (years)	38.2 (2.6)	37.8 (2.6)	38.0 (2.6)
BMI (kg/m^2^)	24.67 (3.91)	25.56 (4.09)	25.11 (4.02)
AMH (ng/ml)	2.48 (2.65)	2.53 (2.54)	2.51 (2.59)
FSH (IU/l)	6.58 (2.43)	6.68 (2.22)	6.63 (2.33)
AFC	15.36 (10.69)	15.03 (9.69)	15.19 (10.18)
Type of infertility, n (%)			
Primary infertility	73 (50.7%)	64 (44.4%)	137 (47.6%)
Secondary infertility	71 (49.3%)	80 (55.6%)	151 (52.4%)
Duration of infertility (years)	3.5 (2.8)	3.2 (2.1)	3.3 (2.4)
Cause of infertility, n (%)			
Female factor	60 (41.7%)	46 (31.9%)	106 (36.8%)
Male factor	39 (27.1%)	45 (31.3%)	84 (29.2%)
Mixed factor	14 (9.7%)	14 (9.7%)	28 (9.7%)
Unexplained infertility	22 (15.3%)	34 (23.6%)	56 (19.4%)
Single women/same-sex couple	9 (6.3%)	5 (3.5%)	14 (4.9%)
Prior IVF cycle, n (%)			
No	80 (55.6%)	71 (49.3%)	151 (52.4%)
Yes	64 (44.4%)	73 (50.7%)	137 (47.6%)
Number of prior IVF cycles	0.7 (1.1)	1.0 (1.6)	0.9 (1.4)
Number of prior embryos transferred	0.7 (1.2)	0.8 (1.4)	0.8 (1.3)

The group characteristics are expressed as mean (SD). AMH, anti-Mullerian hormone; AFC, antral follicular count.

### IVF cycle characteristics

In the ITT analysis, the IVF cycle characteristics were similar between both groups for the following outcomes: total dose of gonadotropins, stimulation days, endometrial thickness on trigger day, baseline IGF-1, IVF cycle outcomes (fresh embryo transfer, freeze all, discard all embryos, failed fertilization, cycle not done, and cycle cancellation), and type of insemination (Table 2).

**Table 2.** IVF cycle characteristics by intention to treat (ITT) analysis.

	GH group (n = 144)	Control group (n = 144)	*P*-value	Mean difference, 95% CI
Total dose of gonadotropins (IU)	4602.9 (1504.9)	4657.1 (1357.3)	0.752	−54.20 [−389.78, 281.38]
Number of stimulation days	11.4 (2.1)	11.7 (1.9)	0.118	−0.30 [−0.77, 0.17]
Endometrial thickness (mm)	10.63 (2.82)	10.94 (2.85)	0.372	−0.31 [−0.99, 0.37]
IGF-1-baseline (ng/nl)	139.6 (42.4)	132.0 (37.3)	0.110	7.61 [−1.69, 16.91]
IGF-1-EoT (ng/nl)	229.6 (72.7)	125.1 (34.6)	<0.00001	104.50 [90.57, 118.43]
IGF-1-ratio (EoT/baseline)	1.71 (0.55)	0.98 (0.24)	<0.00001	0.73 [0.63, 0.83]
(IGF-1-EoT (ng/nl))/(utilizable embryos)	122.5 (87.9)	61.7 (41.1)	<0.00001	60.80 [42.23, 79.37]
IVF cycle outcome n (%)				
Fresh embryo transfer	78 (54.2%)	90 (62.5%)	0.413	
Freeze all embryos	30 (20.8%)	18 (12.5%)		
Discard all embryos (poor quality)	5 (3.5%)	5 (3.5%)		
Failed fertilization	19 (13.2%)	17 (11.8%)		
Cycle not done/excluded	3 (2.1%)	6 (4.2%)		
Cycle cancellation	9 (6.3%)	8 (5.6%)		
Insemination type, n (%)				
Standard IVF	19 (24.4%)	28 (31.1%)	0.718	
ICSI	55 (70.5%)	59 (65.6%)		
Mixed	2 (2.6%)	1 (1.1%)		
PICSI	2 (2.6%)	2 (2.2%)		

The group characteristics are expressed as mean (SD), mean difference with 95% CI. GH, growth hormone; IGF-1-EoT, insulin-like growth factor 1 serum level at the end of treatment; PICSI, physiological intra-cytoplasmic sperm injection.

However, GH injections significantly raised serum IGF-1 levels at the end of treatment in the GH group and similarly, the ratio of end of treatment to baseline IGF-1 was significantly higher in the GH group. Moreover, the IGF-1 level per utilizable embryo in the GH group was nearly double that of the control group (Table 2).

In the PP analysis, results were similar to the ITT population across all parameters (Supplementary Table S2).

### Ovarian stimulation outcomes

In the ITT analysis (Table 3), hormonal levels, follicle count, and embryology results were not statistically different between GH and control groups. The outcomes of the PP analysis paralleled those of the ITT analysis (Table 3).

**Table 3.** Ovarian stimulation outcomes by intention to treat (ITT) and per protocol (PP) analysis.

ITT analysis				
	Growth hormone group (n = 144)	Control group (n = 144)	*P*-value	Mean difference, 95% CI
E2 (pmol/l)	7692.0 (5571.6)	8193.5 (5815.5)	0.472	−501.50 [−1867.79, 864.79]
Progesterone (nmol/l)	2.66 (1.37)	2.77 (1.30)	0.466	−0.11 [−0.43, 0.21]
Number of follicles ≥15 mm	7.8 (5.2)	7.1 (4.2)	0.212	0.70 [−0.41, 1.81]
Number of follicles <15 mm	7.3 (6.4)	7.8 (6.7)	0.556	−0.50 [−2.04, 1.04]
Number of oocytes retrieved	11.7 (8.5)	11.2 (7.9)	0.613	0.50 [−1.49, 2.49]
Number of mature oocytes	8.5 (6.2)	8.6 (6.3)	0.851	−0.10 [−1.62, 1.42]
Maturation rate, %	73.8% (21.6)	78.4% (17.6)	0.060	−4.60 [−9.39, 0.19]
Fertilization rate—all types included, %	64.3% (29.1)	67.2% (25.7)	0.388	−2.90 [−9.59, 3.79]
Number of embryos available for transfer	2.5 (2.4)	2.6 (2.6)	0.767	−0.10 [−0.71, 0.51]

PP analysis				
	Growth hormone group (n = 105)	Control group (n = 105)	*P*-value	Mean difference, 95% CI
E2 (pmol/l)	8122.0 (5550.0)	8827.2 (5975.9)	0.377	−705.20 [−2265.15, 854.75]
Progesterone (nmol/l)	2.70 (1.14)	2.78 (1.18)	0.590	−0.08 [−0.39, 0.23]
Number of follicles ≥15 mm	8.8 (4.9)	8.0 (4.1)	0.192	0.80 [−0.42, 2.02]
Number of follicles <15 mm	8.2 (6.7)	8.4 (6.8)	0.846	−0.20 [−2.03, 1.63]
Number of oocytes retrieved	12.4 (7.9)	12.0 (8.2)	0.681	0.40 [−1.78, 2.58]
Number of mature oocytes	9.2 (6.2)	9.3 (6.5)	0.974	−0.10 [−1.82, 1.62]
Maturation rate, %	76.0% (17.6)	78.9% (17.0)	0.232	−2.90 [−7.58, 1.78]
Fertilization rate—all types included, %	69.3% (23.9)	70.9% (22.6)	0.625	−1.60 [−7.89, 4.69]
Number of embryos available for transfer	3.0 (2.4)	3.0 (2.4)	0.954	0.00 [−0.65, 0.65]

The group characteristics are expressed as mean (SD), mean difference with 95% CI. E2: serum estradiol level at the day of trigger. Progesterone: serum progesterone level at the day of trigger.

### Reproductive outcomes

In fresh embryo transfer cycles (n = 78), the rates of Day 3 embryo transfers were 57.7% for the GH group and 56.7% for the control group, while Day 5–6 embryo transfers were 42.3% for GH and 43.3% for controls. There were no statistically significant differences between groups in any of the outcome measures (Table 4).

**Table 4.** Reproductive outcomes in fresh embryo transfer cycles.

Fresh embryo transfer	Growth hormone group (n = 78)	Control group (n = 90)	*P*-value	Mean or rate difference, 95% CI
Embryo stage at transfer (fresh), n (%)				
Day 3 embryo	45 (58%)	51 (57%)	0.893	
Day 5–6 embryo	33 (42%)	39 (43%)		
Implantation rate, mean % (SD)	38.2% (47.6)	39.8% (46.3)	0.829	−1.60 [−15.85, 12.65]
Clinical pregnancy rate,	34 (44%)	45 (50%)	0.406	−0.06 [−0.22, 0.09]
Miscarriage rate	9 (27%)	14 (31%)	0.653	−0.05 [−0.25, 0.15]
Live birth rate	25 (32%)	30 (33%)	0.860	−0.01 [−0.15, 0.13]
Overall average number of fresh embryos transferred per clinical pregnancy (total transferred/total clinical pregnancies)	3.0 (102/34)	2.5 (114/45)		

The group characteristics are expressed as n (%) unless stated otherwise, mean difference or rate difference shows value with 95% CI.

Regarding the first cycle of FETs (n = 57), 36.8% of the GH group had Day 3 embryos transferred compared to 25.6% in the control group, and 63.2% of GH group had Day 5–6 embryos versus 74.4% in controls. The implantation rate was 31.6% for GH and 45.3% for controls (*P* = 0.178, mean difference, 95% CI: −13.70 [−34.01, 6.61]); clinical pregnancy rates were 35.1% for GH and 44.2% for controls (*P* = 0.356, rate difference, 95% CI: −0.09 [−0.28, 0.10]); relative rate, 95% CI: 0.79 [0.49, 1.29]); the miscarriage rates came in at 28.6% for GH and 26.3% for controls (*P* = 0.873, rate difference, 95% CI: 0.02 [−0.25, 0.30]); and live birth rates were 22.8% for GH compared to 32.6% for controls (*P* = 0.277, rate difference, 95% CI: −0.10 [−0.27, 0.08]). Again, no statistical difference was found for these parameters. The GH group had an average of 3.1 embryos transferred per clinical pregnancy compared to 2.4 for the control group (Table 5).

**Table 5.** Reproductive outcomes in first frozen embryo transfer cycles.

First frozen embryo transfer	GH group (n = 76)	Control group (n = 69)	*P*-value	Mean or rate difference, 95% CI
Embryo stage at transfer (frozen), n (%)				
Day 3 embryo	21 (37%)	11 (26%)	0.232	
Day 5–6 embryo	36 (63%)	32 (74%)		
Implantation rate, mean % (SD)	31.6% (46.9)	45.3% (54.4)	0.178	−13.70 [−34.01, 6.61]
Clinical pregnancy rate, % (n)	20 (35%)	19 (44%)	0.356	−0.09 [−0.28, 0.10]
Miscarriage rate, % (n)	6 (29%)	5 (26%)	0.873	0.02 [−0.25, 0.30]
Live birth rate, % (n)	13 (23%)	14 (33%)	0.277	−0.10 [−0.27, 0.08]
Overall average number of frozen embryos transferred per clinical pregnancy (total transferred/total clinical pregnancies)	3.1 (62/20)	2.4 (46/19)		

The group characteristics are expressed as n (%) unless stated otherwise, mean difference or rate difference shows value with 95% CI. GH, growth hormone.

## Discussion

Giving GH therapy to all IVF patients, regardless of ovarian reserve or past reproductive outcomes, has been previously proposed. The idea was examined in two RCTs in 1992 (Tapanainen *et al.*, 1992; Younis *et al.*, 1992), involving a small number of patients. Unfortunately, recent, reliable evidence about the universal use of GH in ART remains lacking. Our study, with a sample size of 288 randomized patients, is thus the largest RCT to date. Similar to earlier RCTs targeting the general IVF population, our results found no clinical advantage from routine use of GH. In addition to the number of participants, our study followed a rigorous randomization process, strengthening the credibility and applicability of its findings.

The role of GH in human reproduction is revealed by the fact that GH deficiency in eugonadotropic patients is linked to infertility, with treatments showing improved outcomes (Yovich *et al.*, 2019). To prevent bias from GH-deficient patients with low IGF-1, we ensured the accuracy of our conclusion by measuring serum IGF-1 both before and after GH therapy. Such measurements are typically absent in most related published articles (Tapanainen *et al.*, 1992; Younis *et al.*, 1992; Liu *et al.*, 2019). These measurements add to the depth of data collected, allowing for a better understanding of GH’s impact, and more importantly open the door for more nuanced studies in the future for individualized GH therapy.

Despite its strengths, this study did not examine long-term outcomes or the effect of repeated cycles with GH. While clinical pregnancy is a significant milestone in IVF treatment, the ultimate goal is achieving a live birth. We acknowledge the importance of considering the live birth rate as a primary outcome. However, obtaining data on live birth rates requires additional years of follow-up and data collection, which is an ongoing process. Until then, we recognize that the clinical pregnancy rate remains an informative outcome measure.

The dropout rate after the completion of the study was 27%, higher than anticipated. This was largely due to patients’ desire to receive GH therapy. The extended length of recruitment is a reflection of the strict design and thorough screening. Initially, 528 patients were determined to be needed for adequate statistical power, based on a 40% increase in the clinical pregnancy rate. After revising the sample size based on a 50% increase in the clinical pregnancy rate, 288 patients were deemed required for recruitment. This recalculation made the project viable considering the strict recruitment criteria that were expected to make recruitment challenging. Consequently, we managed to obtain clinically relevant results with this sample size. The revised sample size was approved by Health Canada and the ethics committee prior to the commencement of patient recruitment. Regarding the open-label design, it was mainly due to financial reasons driven by the expensive additional cost of preparing sham injections for the control group. Nevertheless, the study’s scientific integrity was respected despite the dropout rate, the extended timeline, and the open-label design. This was maintained through rigorous randomization and screening methods.

Our study confirms previous findings in normal responders (Tapanainen *et al.*, 1992; Younis *et al.*, 1992). The recent case-control study differs from ours, given its focus on patients with a prior poor reproductive outcome (Liu *et al.*, 2019). Almost half of our patients in both arms had previous IVF experience. However, in our study, we did not collect detailed information on the outcomes of prior cycles. Thus, we did not stratify patients based on the results of previous IVF.

Nowadays, there is a growing trend toward individualized treatment in IVF. GH therapy has been studied with possible benefits for poor responders (Sood *et al.*, 2021), poor oocyte quality (Gong *et al.*, 2020; Tesarik *et al.*, 2020; Scheffler *et al.*, 2021), poor embryo quality (Liu *et al.*, 2019), recurrent implantation failure (Chen *et al.*, 2018; Hart, 2019), and thin endometrium (Cui *et al.*, 2019; Liu *et al.*, 2019). Although GH therapy in IVF might benefit certain groups, our study found no advantage for routine GH supplementation in IVF.

## Conclusion

GH adjuvant therapy in GnRH antagonist cycles does not improve IVF stimulation results or reproductive outcomes, specifically implantation, miscarriage, and clinical pregnancy rates in the general IVF population.

## Supplementary Material

deae251_Supplementary_Table_S1

deae251_Supplementary_Table_S2

## Data Availability

The data underlying this article will be shared on reasonable request to the corresponding author.
