# Supplementary material for: Empirical use of growth hormone in IVF is useless: the largest randomized controlled trial
Source: Hum Reprod. 2024 Nov 29;40(1):77–84. doi: 10.1093/humrep/deae251 (PMC11700900; doi:10.1093/humrep/deae251)
Supplement: deae251_Supplementary_Table_S1 [file deae251_supplementary_table_s1.pdf]

OXFORD
human reproduction
*Human Reproduction*, 2024, 1–1
https://doi.org/10.1093/humrep/deae251
Supplementary Data

**Supplementary Table S1.** Vitamins and supplements intake in the intervention (growth hormone [GH]) and control groups.

| | GH group (n = 144) n (%) | Control group (n = 144) n (%) | Overall (n = 288) n (%) |
|---|---|---|---|
| Dehydroepiandrosterone | 46 (31.9%) | 33 (22.9%) | 79 (27.4%) |
| Fertil-Pro-LQ | 34 (23.6%) | 36 (25.0%) | 70 (24.3%) |
| L-arginine | 1 (0.7%) | 1 (0.7%) | 2 (0.7%) |
| Fertil-Pro for women | 52 (36.1%) | 42 (29.2%) | 94 (32.6%) |
| Pregnancy vitamins | 15 (10.4%) | 9 (6.3%) | 24 (8.3%) |
| Folic acid | 65 (45.1%) | 54 (37.5%) | 119 (41.3%) |
| Vitamin D | 48 (33.3%) | 40 (27.8%) | 88 (30.6%) |
