# Supplementary material for: Empirical use of growth hormone in IVF is useless: the largest randomized controlled trial
Source: Hum Reprod. 2024 Nov 29;40(1):77–84. doi: 10.1093/humrep/deae251 (PMC11700900; doi:10.1093/humrep/deae251)
Supplement: deae251_Supplementary_Table_S2 [file deae251_supplementary_table_s2.pdf]

OXFORD
human reproduction

Human Reproduction, 2024, 1–1
https://doi.org/10.1093/humrep/deae251
Supplementary Data

Supplementary Table S2. IVF cycle characteristics by per protocol analysis.

	Growth hormone group (n = 105)	Control group (n = 105)	P-value	Mean difference, 95% CI
Total dose of gonadotropins (IU)	4542.8 (1371.0)	4582.3 (1202.7)	0.825	−39.50 [−388.34, 309.34]
Number of stimulation days	11.6 (1.7)	11.8 (1.5)	0.318	−0.20 [−0.63, 0.23]
Endometrial thickness (mm)	10.72 (2.40)	11.06 (2.70)	0.348	−0.34 [−1.03, 0.35]
IGF-1-EoT (ng/nl)	225.1 (69.8)	121.6 (33.4)	<0.00001	103.50 [88.47, 118.53]
IGF-1-ratio (EoT/baseline)	1.68 (0.53)	0.97 (0.24)	<0.00001	0.71 [0.60, 0.82]
(IGF-1-EoT (ng/nl))/(utilizable embryos)	123.0 (88.8)	63.0 (41.1)	<0.00001	60.00 [41.00, 79.00]
IVF cycle outcome				
Fresh embryo transfer	78 (74.3%)	90 (85.7%)	0.038	
Freeze all embryos	27 (25.7%)	15 (14.3%)		
Insemination type, n (%)				
Standard IVF	19 (24.4%)	28 (31.1%)	0.718	
ICSI	55 (70.5%)	59 (65.6%)		
Mixed	2 (2.6%)	1 (1.1%)		
PICSI	2 (2.6%)	2 (2.2%)		

The group characteristics are expressed as mean (SD), mean difference with 95% CI. IGF-1, insulin-like growth factor 1; EoT, end of treatment; PICSI, physiological intra-cytoplasmic sperm injection.
